# Detection of Novel Thermotolerant *Tepidimonas* sp. Bacteria in Human Respiratory Specimens, Hong Kong, China, 2024

**DOI:** 10.3201/eid3201.250818

**Published:** 2026-01

**Authors:** Kelvin Hei-Yeung Chiu, Shuk-Ching Wong, Edwin Kwan-Yeung Chiu, Raymond Hon Man Ng, Jonathan Hon-Kwan Chen, Jordan Yik-Hei Fong, Lithia Lai-Ha Yuen, Simon Yung-Chun So, David Christopher Lung, Vincent Chi-Chung Cheng, Kwok-Yung Yuen, Jade Lee-Lee Teng

**Affiliations:** Queen Mary Hospital, Hong Kong, China (K.H.-Y. Chiu, S.-C. Wong, E.K.-Y. Chiu, R.H.-M. Ng, J.H.-K. Chen, L.L.-H. Yuen, S.Y.-C. So, V.C.-C. Cheng); University of Hong Kong, Hong Kong (S.-C. Wong, J.H.-K. Chen, J.Y.-H. Fong, K.-Y. Yuen, J.L.-L. Teng); Queen Elizabeth Hospital, Hong Kong (D.C. Lung); Hong Kong Children’s Hospital, Hong Kong (D.C. Lung)

**Keywords:** bacteria, Tepidimonas, pneumonia, respiratory infections, Hong Kong, China

## Abstract

In patients with acute respiratory infections (ARIs), routine diagnostic tests often fail to identify the microbial cause; thus, many ARIs have undetermined etiology. We investigated potential involvement of thermotolerant bacteria in ARIs among patients in Hong Kong, China, by incubating blood agar inoculated with respiratory specimens at 50°C for 5 days. Among 7,257 specimens analyzed, 58 specimens from 57 patients grew thermotolerant bacteria not identified by other methods. We identified *Tepidimonas* spp. in 42 isolates, 3 of which appear to be a novel *Tepidimonas* species (tentatively *Tepidimonas hongkongensis* sp. nov). Genomic analysis revealed various virulence, resistance, and stress-related genomes in the 3 isolates. *Tepidimonas* spp. bacteria were predominantly isolated from patients with chronic lung disease and malignancies. We also detected *T. hongkongensis* in hospital water samples but at a lower percentage than in respiratory specimens, suggesting colonization potential. Clinical implications of *T. hongkongensis* remain unknown; continued surveillance could determine its role in ARIs.

Although routine bacterial culture and multiplex reverse transcription PCR for respiratory viruses and fastidious organisms are commonly performed in respiratory specimens from patients with acute respiratory syndrome, the distinct microbial cause remains unidentified in most patients. A study from China showed that among school-age children with acute respiratory infections (ARIs), 46.9% of viral and 30.9% of bacterial etiologies could be identified, but the other patients had ARIs of unknown etiology (*1*). 

The routine practice of bacterial culture of expectorated sputum relies on blood and chocolate agar plates incubated at 35°C–37°C for 48 hours. Those methods could miss bacteria that grow poorly or very slowly at body temperature and might be overgrown by common oropharyngeal commensals. Although most respiratory pathogens are not thermotolerant, pathogens such as *Aspergillus* spp. fungi and the bacterium *Mycobacterium xenopi* are thermotolerant and have been described as causing respiratory infections, especially in patients with chronic lung disease (*2*).

We hypothesized that increasing the incubation temperature during culture can suppress normal flora and enable fastidious, thermotolerant bacteria to grow, possibly revealing novel pathogens contributing to ARIs. We investigated respiratory specimens by prolonged incubation of agar plates at high temperature, then subjected cultured bacterial isolates to matrix-assisted laser desorption/ionization time-of-flight (MALDI-TOF) mass spectrometry and partial 16S rRNA gene sequencing to determine whether thermotolerant bacteria were the cause of ARIs.

## Materials and Methods

### Study Design and Specimen Collection

We conducted a multicenter, retrospective study in a hospital network in Hong Kong, China, including a 1,700-bed university-affiliated teaching hospital (Queen Mary Hospital) and 3 extended care hospitals ranging in size from 270 to 530 beds. We included all respiratory specimens, including sputum and bronchoalveolar lavage fluid (BALF) samples, collected during January 19–May 19, 2024, and sent to the microbiology laboratory at Queen Mary Hospital for routine bacterial culture. This study was approved by the Institutional Review Board of the University of Hong Kong Special Administrative Region, People’s Republic of China/Hospital Authority Hong Kong Special Administrative Region, People’s Republic of China West Cluster (UW 25-135).

### Routine Investigations Methodology

As part of routine screening, we performed Gram stains on all lower respiratory specimens to semiquantify the number of leukocytes and assess specimen quality. We observed Gram stains under light microscopy at ×100 magnification and graded stains on the basis of the number of leukocytes as trace (<25 cells), 2+ (25–75 cells), or 3+ (>75 cells) and epithelial cells as 1+ (<10 cells), 2+ (10–25 cells), or 3+ (>25 cells) in an average of 10 fields. We inoculated all samples on blood and chocolate agar plates for routine bacterial culture and also inoculated BALF samples on MacConkey agar plates. Plates routinely are incubated at 35°C at 5% CO_2_ and examined every 24 hours for 2 days. For this study, we also inoculated all respiratory tract samples on separate blood agar plates, incubated at 50°C, and examined plates every 24 hours for 5 days. We defined any colonies that grew from the blood agar plates at 50°C as thermotolerant bacteria, and subjected those to MALDI-TOF spectrometry and 16S rRNA PCR (*3*), when necessary, for bacterial identification. If the MALDI-TOF In Vitro Diagnostic database (Bruker, https://www.bruker.com) failed to identify bacteria, we performed subsequent Sanger sequencing. 

### 16S rRNA, MALDI-TOF Spectrometry, and Whole-Genome Sequencing Methods

When MALDI-TOF spectrometry failed to identify bacterial isolates, we performed 16S rRNA sequencing. We used universal bacterial primers 16S-1F and 16S-2R and the QIAmp DNA Minikit (QIAGEN, https://www.qiagen.com) to extract DNA and sequenced on the 3500 Genetic Analyzer system (Thermo Fisher Scientific, https://www.thermofisher.com) (Appendix). 

We considered bacteria that had suboptimal similarity percentages with existing bacteria in the BLAST (https://blast.ncbi.nlm.nih.gov) as possible novel species (*4*). We subjected those bacteria to whole-genome sequencing by using a dual platform of short- and long-read sequencing. For short-read sequencing, we performed DNA preparation by using the Nextera DNA Prep Kit, then sequenced on the iSeq100 (Illumina, https://www.illumina.com). For long-read sequencing, we used the Ligation Sequencing Kit for DNA preparation and MinION for sequencing (both Oxford Nanopore Technologies, https://nanoporetech.com) (Appendix). 

### Covariates of Interest

We retrieved data on patients who had thermotolerant bacterial species identified from electronic health records. Patient information included demographic data, underlying medical conditions, signs and symptoms, time from hospital admission to sample collection, hematologic parameters, inflammatory markers, and radiologic findings.

### Statistical Analysis

We reported descriptive statistics as median and range and categorical variables as frequency and percentage. We used Fisher exact or χ^2^ tests for 2-group comparisons. We performed statistical analyses in SPSS Statistics 24.0 (IBM, https://www.ibm.com) and considered p<0.05 statistically significant.

## Results

During January 19–May 19, 2024, the microbiology laboratory collected 7,257 respiratory specimens, of which 6,766 were lower respiratory tract specimens: 5,922 expectorated sputum, 388 tracheal aspirates, and 456 bronchial specimens, including BALF and endobronchial biopsy specimens. The other 491 respiratory specimens were upper respiratory tract specimens: 113 nasal or nasopharyngeal swab samples, 283 oral or throat swab samples, and 95 gastric aspirates.

Among collected specimens, 66 (64 sputum and 2 throat swab) specimens from 65 patients grew thermotolerant bacteria that could not be identified by MALDI-TOF spectrometry (Table 1). Among the 66 thermotolerant bacterial specimens, we identified *Tepidimonas* spp. in 42 (63.6%, 0.6% of all respiratory specimens, 41 sputum and 1 throat swab) specimens from 41 patients by 16S rRNA sequencing; 37 were *T. aquatica*, 3 were undetermined *Tepidimonas* spp., 1 was *T. taiwanensis*, and 1 was *T. fonticaldi.*

**Table 1:** Bacteria from respiratory specimens collected in a study on detection of novel thermotolerant *Tepidimonas* spp. bacteria in human respiratory specimens, Hong Kong, China, 2024*

Thermotolerant bacteria	No. (%) specimens
*Tepidimonas* spp.	42 (63.6)
* T. aquatica*	37
* T. taiwanensis*	1
* T. fonticaldi*	1
Unidentified *Tepidimonas* spp.†	3
*Schlegelella aquatica*	14 (12.1)
*Vulcaniibacterium thermophilum*	4 (6.1)
*Thermomonas* spp.	2 (3.0)
*Lactobacillus delbrueckii*	1 (1.5)
*Bacillus* species	3 (4.5)
* B. gelatini*	1
* B. licheniformis*	1
* B. safensis*	1

*Identified by matrix-assisted laser desorption/ionization time-of-flight mass spectrometry. †Unable to identify species-level by 16S rRNA sequencing.

Patients with *Tepidimonas* spp. bacterial isolates were from all age groups; 56.1% of patients were female and 43.9% were male (Table 2). The most common underlying conditions in the cohort were solid organ malignancy (41.5%), hypertension (36.6%), chronic lung disease (24.4%), and hyperlipidemia (24.4%). Among *Tepidimonas* spp. isolates, 58.5% were from sputum saved in the outpatient setting or collected <48 hours of hospital admission, suggesting a possible community source. Common clinical signs and symptoms included cough, fever, and shortness of breath. Some of the patients had abnormal chest radiograph findings, including consolidation or pulmonary infiltrates (26.8%), pleural effusion (19.5%), and chronic lung changes (24.4%). However, 34.1% of patients with *Tepidimonas* spp. isolated had unremarkable findings on chest radiographs, representing possible bacterial colonization of the respiratory tract.

**Table 2:** Demographic characteristics of 41 patients from whom *Tepidimonas* spp. bacteria were detected in respiratory specimens, Hong Kong, China, 2024*

Characteristics	Value
Median age, y (range)	70 (15–97)
Sex	
F	23 (56.1)
M	18 (43.9)
Underlying conditions	
Hypertension	15 (36.6)
Diabetes mellitus	7 (17.1)
Hyperlipidemia	10 (24.4)
Cardiovascular disease	9 (22.0)
Renal impairment	7 (17.1)
Cerebral vascular accident	1 (2.4)
Chronic lung disease	10 (24.4)
Chronic liver disease	7 (17.1)
Solid organ malignancy	17 (41.5)
Hematologic malignancy	1 (2.4)
Autoimmune disease	1 (2.4)
Timing of positive growth	
Outpatient or <48 h of admission	24 (58.5)
>48 h after admission	17 (41.5)
Clinical signs and symptoms	
Fever	13 (31.7)
Cough	14 (34.1)
Sputum	11 (26.8)
Shortness of breath	13 (31.7)
Chest radiography	
Not done	2 (4.9)
Unremarkable	14 (34.1)
Consolidation or pulmonary infiltrates	11 (26.8)
Pleural effusion	8 (19.5)
Chronic lung changes	10 (24.4)
Blood parameters, median (range)	
Leukocytes, × 10^9^ cells/L	8.34 (1.77–23.10)
Neutrophils, × 10^9^ cells/L	5.94 (1.29–20.95)
C-reactive protein, mg/dL	5.21 (0.17–18.53)

*Values are no. (%) except as indicated.

Among laboratory characteristics of the 41 sputum specimens, 51.2% had trace leukocytes, and 46.3% had <10 epithelial cells per field (Table 3). In addition, 36.6% had growth of other pathogenic organisms, the most common of which were *Haemophilus influenzae* and *Flavobacterium* species.

**Table 3:** Characteristics of isolate cultures from 41 sputum specimens in study of novel thermotolerant *Tepidimonas* spp. bacteria in human respiratory specimens, Hong Kong, China, 2024

Characteristics	No. (%)
Leukocyte quantified by Gram stain	
Negative	7 (17.1)
Trace	21 (51.2)
2+	7 (17.1)
3+	6 (14.6)
Epithelial cells quantified by Gram stain	
Negative	4 (9.8)
1+	19 (46.3)
2+	10 (24.4)
3+	8 (19.5)
Other organisms grown	15 (36.6)
*Acinetobacter* species	2 (4.9)
*Candida* species	2 (4.9)
* Escherichia coli*	1 (2.4)
*Flavobacterium* species	3 (7.3)
* Haemophilus influenzae*	4 (9.8)
* Pseudomonas aeruginosa*	2 (4.9)
Methicillin-resistant *Staphylococcus aureus*	1 (2.4)
* Stenotrophomonas maltophilia*	2 (4.9)

### Novel Species Identification

Our study identified 3 clinical isolates of a novel *Tepidimonas* sp. bacterium. Here, we report phenotypic characteristics and genomic features of those bacteria, including detection of virulence, antimicrobial resistance (AMR), and stress-related genes. To investigate potential sources of this bacterium, we also performed environmental sampling in the hospital.

#### Phenotypic Analysis and Biochemical Characteristics

The 3 undetermined sequences detected by 16S rRNA sequencing displayed 96.68%–97.59% sequence identity to known *Tepidimonas* spp. available in the BLAST database, suggesting that the taxonomic position of those isolates remained uncertain. The sequences were from 3 isolates (HKU77, HKU78, and HKU79) from 3 patients: 1 with pulmonary metastasis, 1 with community-acquired pneumonia, and 1 with underlying Graves’ disease and schizophrenia who had fever during hospitalization.

Gram stain of isolates HKU77, HKU78, and HKU79 revealed gram-negative, non–spore-forming, motile rods with occasional spherical enlargement (Appendix Figure, panel A). We observed small translucent and nonpigmented colonies with no hemolysis on blood agar after 24 hours of incubation. The size of colonies gradually increased with prolonged incubation. We noted no pigment production at 35°C or 42°C, but colonies displayed a slight brown pigmentation after prolonged incubation at 50°C. Optimal growth of those bacteria occurred at 50°C, and we noted no visible growth at 25°C (Appendix Figure, panels B–D). The 3 isolates did not grow in anaerobic conditions. Growth on chocolate agar and *Haemophilus* test medium at 50°C was similar to that for blood agar; we observed slower growth on *Brucella* agar. However, we noted no growth on buffered charcoal yeast extract agar or brain heart infusion agar supplemented with X and V factor. We observed no fluorescence under ultraviolet light (Table 4).

**Table 4:** Laboratory characteristics of 3 strains of novel thermotolerant *Tepidimonas* spp. bacteria isolated in human respiratory specimens, Hong Kong, China, 2024*

Test	HKU77^T^	HKU78	HKU79
Gram stain	Gram-negative rod	Gram-negative rod	Gram-negative rod
Anaerobic growth	No growth	No growth	No growth
Motility	Motile	Motile	Motile
Oxidase	Positive	Positive	Positive
Catalase, 15% H_2_O_2_	Weakly positive	Weakly positive	Weakly positive
Yellow pigment	Slightly brown	Slightly brown	Slightly brown
Hemolysis	Negative	Negative	Negative
UV fluorescence	Negative	Negative	Negative
Growth, 72 h			
25°C	No growth	No growth	No growth
37°C	Very slow growth	Very slow growth	Very slow growth
42°C	Slow growth	Slow growth	Slow growth
50°C	Growth	Growth	Growth
Growth rate, 50°C, 72 h			
Chocolate agar	Same as blood agar	Same as blood agar	Same as blood agar
*Haemophilus* test medium	Nearly same as blood agar	Nearly same as blood agar	Nearly same as blood agar
Brucella agar	Slower than blood agar	Slower than blood agar	Slower than blood agar
BCYE agar	No growth	No growth	No growth
Oxoid brain–heart infusion agar	No growth	No growth	No growth
Egg yolk agar, 50°C	Negative	Negative	Negative
DNAase agar, 50°C	Negative	Negative	Negative
Gelatinase agar, 50°C	Negative	Negative	Negative
Glucose oxidation and fermentation test, 50°C	Inert	Inert	Inert
Nitrate, 50°C	Positive	Positive	Positive
Urease, 50°C	Weakly positive	Weakly positive	Weakly positive
ONPG, 50°C	Negative	Negative	Negative
Positive by VITEK AutoMicrobic GNI+ system†, McFarland 2	Glutamyl arylamidsae, tyrosine arylamidase	Glutamyl arylamidsae, tyrosine arylamidase, L-proline arylamidase, Ellman’s test	Glutamyl arylamidsae, tyrosine arylamidase
MIC, μg/mL at 50°C			
Vancomycin	64	48	64
Penicillin	<0.016	<0.016	<0.016

*Strains of novel *T. hongkongensis*. BCYE, buffered charcoal yeast extract; ONPG, ortho-Nitrophenyl-β-galactoside; UV, ultraviolet. †bioMérieux, https://www.biomerieux.com.

Biochemically, all 3 isolates were oxidase-positive and weakly catalase-positive (with 15% hydrogen peroxide) and tested negative for lipase, lecithinase, DNAase, and gelatinase at 50°C. The isolates were positive for nitrate reductase, glutamyl arylamidase, and tyrosine arylamidase and weakly positive for urease. Sugar fermentation (glucose, lactose, sucrose, citrate), indole, H_2_S production, and ortho-nitrophenyl-β-galactoside were all negative after incubation at 50°C. We evaluated enzymatic activity by using the VITEK AutoMicrobic GNI+ card (bioMérieux, https://www.biomerieux.com), which was positive for glutamyl arylamidase and tyrosine arylamidase, and 1 strain (HKU78) showed additional enzymatic activity of L-proline arylamidase and a positive Ellman’s test result. 

The 3 isolates were susceptible to piperacillin, piperacillin/tazobactam, ceftazidime, amikacin, ciprofloxacin, tobramycin, and cefepime, according to the Clinical Laboratory Standards Institute *Pseudomonas* breakpoints (*5*). The MICs of penicillin for the 3 isolates were <0.016 μg/mL, and MICs of vancomycin were 48–64 μg/mL. Comparison with phenotypic characteristics of *T. aquatica* and *T. taiwanensis* showed that the *Tepidimonas* species from our 3 strains did not grow on buffered charcoal yeast extract agar, but both *T. aquatica* and *T. taiwanensis* did. Furthermore, *T. taiwanensis* is gelatinase- and nitrate-positive, but *T. aquatica* and the novel *Tepidimonas* species from our study are not (Table 4).

#### Comparative Genomic Characterizations

**Figure 1:**
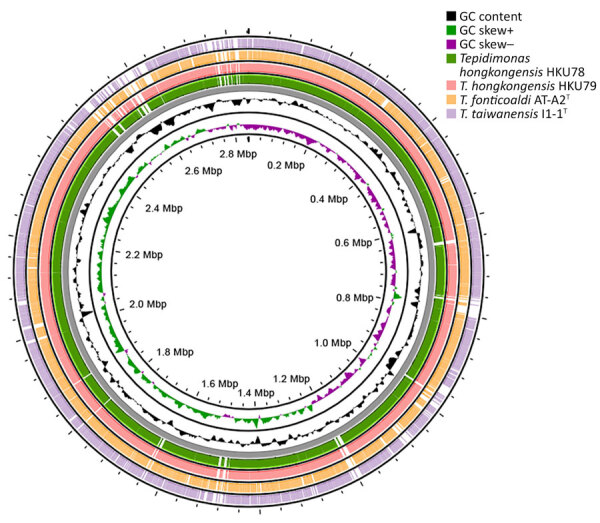
Circular genome map of novel thermotolerant *Tepidimonas* spp. bacteria detected in human respiratory specimens, Hong Kong, China, 2024. Using patient strain HKU77^T^ as the reference genome forming the backbone, the ring compares genome matches from patient strains HKU78 and HKU79 and the 2 closest *Tepidimonas* species, *T. fonticoaldi* AT-A2^T^ and *T. taiwanensis* I1–1^T^ genomes. The genome features include GC content and plus (+) or minus (–) GC skew.

**Figure 2:**
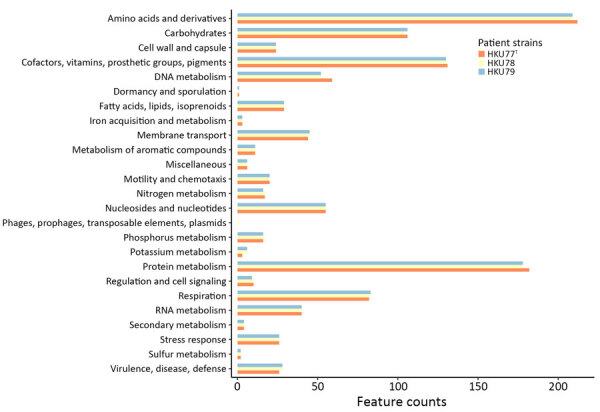
Comparison of feature counts of functional annotations from detection of novel thermotolerant *Tepidimonas* spp. bacteria in human respiratory specimens, Hong Kong, China, 2024. Annotations were determined by using Rapid Annotations using Subsystems Technology (Argonne National Laboratory, https://www.anl.gov/mcs/rast-rapid-annotation-using-subsystem-technology). Each functional category is represented by bars corresponding to the 3 patient strains of *T. hongkongensis* identified in this study.

The de novo assembly, using Illumina and Oxford Nanopore Technology reads, yielded complete genomes for the strains HKU77, HKU78, and HKU79 (Figure 1). The genome sizes of the 3 strains ranged from 2,842,968 to 2,891,522 bp, and GC content spanned 67.1%–67.2% (Table 5). We submitted the assembled genome sequences to PROKKA (https://github.com/tseemann/prokka) for annotation, which resulted in 2,695–2,786 protein-coding sequences, 3 rRNA operons, and 50–52 tRNA-coding genes (Table 5). Annotation via the RAST pipeline (Rapid Annotations using Subsystems Technology; Argonne National Laboratory, https://www.anl.gov/mcs/rast-rapid-annotation-using-subsystem-technology) indicated that the 3 strains possess a similar number of genes and subsystems, except for strain HKU78, which harbored a phage genome containing an additional integron integrase *IntI1* gene related to the phages, prophages, transposable elements, and plasmids subsystem in RAST (Figure 2).

**Table 5:** Genome characteristics and functional annotation in study of novel thermotolerant *Tepidimonas* spp. bacteria in human respiratory specimens, Hong Kong, China, 2024*

Features	HKU77^T^	HKU78	HKU79
Size, bp	2,842,968	2,875,687	2,891,522
No. contigs	1	1	1
% GC	67.2	67.1	67.2
No. coding sequences	2,695	2,744	2,786
No. rRNA sequences	3	3	3
No. tRNA sequences	51	52	50

*Genome statistics obtained from PROKKA (https://github.com/tseemann/prokka) annotation and seqkit (https://github.com/shenwei356/seqkit/releases) results.

**Figure 3:**
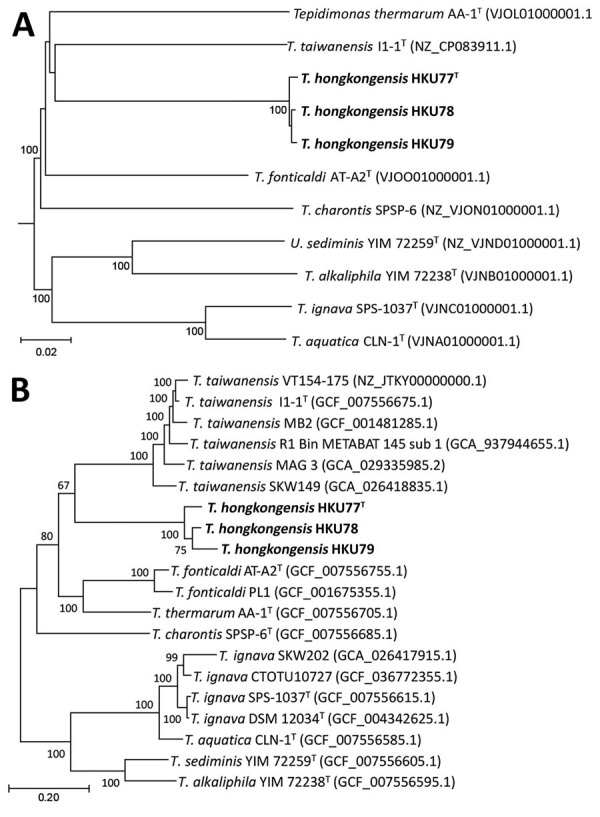
Phylogeny of novel thermotolerant *Tepidimonas* spp. bacteria isolated from human respiratory specimens, Hong Kong, China, 2024. Phylogenetic trees show the relationship between 3 patient strains identified in this study (bold font) and global representative *Tepidimonas* spp. strains. A) Tree was inferred using whole-genome data with Type (Strain) Gene Server (Leibniz Institute, https://tygs.dsmz.de). B) Tree inferred from the species-specific core genes and compared with existing *Tepidimonas* spp. in GenBank. Scale bar indicates nucleotide substitutions per site.

To determine the phylogenetic positions of the 3 strains within the genus *Tepidimonas*, we used Type (Strain) Gene Server (Leibniz Institute, https://tygs.dsmz.de) results to construct a phylogenetic tree incorporating genome data from the 3 strains and complete genomes of other *Tepidimonas* spp. type strains. That analysis revealed that HKU77, HKU78, and HKU79 cluster together, forming a distinct and well-supported phylogenetic clade, separate from other *Tepidimonas* species, and are most closely related to *T. fonticaldi* (Figure 3, panel A). Similarly, the phylogenetic tree constructed from core-genome sequences displayed a consistent topology (Figure 3, panel B). In silico genome-to-genome comparison demonstrated that the 3 strains from our study shared pairwise digital DNA-DNA hybridization (dDDH) values ranging from 95.3% to 97.8% among each other. Those 3 strains were most closely related to *T. fonticaldi* AT-A2 (dDDH value 26.1%–26.2%), followed by *T. taiwanensis* I1–1 (dDDH value 24.7%–24.8%), and *T. charontis* SPSP-6T (dDDH value 24.0%–24.3%), but the dDDH values were below the 70.0% threshold, indicating the 3 strains from our study are a different species (Table 6). On the basis of those results, we propose that strains HKU77, HKU78, and HKU79 represent a newly identified *Tepidimonas* species. We suggest the name *Tepidimonas hongkongensis* sp. nov. for the location of isolation and that strain HKU77^T^ (GenBank accession no. CP187300) be designated as the type strain.

**Table 6:** Pairwise genomic alignments in study of novel thermotolerant *Tepidimonas* spp. bacteria in human respiratory specimens, Hong Kong, China, 2024*

*Tepidimonas* species	ID	Genome size, bp	GC, %	No. proteins	No. rRNA	No. tRNA	% d4
*T. hongkongensis* reference	HKU77^T^	2,842,968	67.2	2,695	3	51	NA
*T. hongkongensis *	HKU78	2,924,694	67.1	2,807	6	52	95.3
*T. hongkongensis *	HKU79	2,891,522	67.2	2,786	3	50	95.5
*T. fonticaldi *	AT-A2	3,009,526	69.0	2,761	6	54	26.1
*T. taiwanensis *	I1–1	2,915,587	68.8	2,671	9	56	24.7
*T. charontis *	SPSP-6T	2,808,982	66.6	2,634	8	55	24.0
*T. thermarum *	AA1	2,703,753	68.7	2,552	6	51	23.8
*T. aquatica *	CLN-1	2,672,904	68.6	2,506	4	58	23.4
*T. ignava *	DSM 12034	2,715,700	68.8	2,562	6	54	22.7
*T. sediminis *	YIM 72259	2,533,936	71.8	2,337	5	58	22.5
*T. alkaliphila *	YIM 72238	2,465,445	69.0	2,279	3	54	21.8

*Comparison through digital DNA-DNA hybridization by using Type (Strain) Gene Server (Leibniz Institute, https://tygs.dsmz.de). ID, identification.

#### Virulence, AMR, and Stress-Related Genes

We characterized virulence factors of *T. hongkongensis* by using the Virulence Factors of Pathogenic Bacteria database (https://www.mgc.ac.cn/VFs/main.htm), on the basis of available sequences (Appendix Table 1). We identified several genes that contribute to the function of motility and adherence, some of which are similar to genes of type IV pili of *Pseudomonas* spp., including *pilB*, *pilC*, and *pilT*, contributing to the adhesion of *T. hongkongensis* to other cells. We also detected different genes related to immune evasion and several genes that contribute to counteracting phagocytosis in humans. Those genes included *uge*, which is observed to generate the capsule in *Klebsiella* spp. Bacteria, and *algC*, *algU*, and *algW*, which are responsible for alginate regulation and biosynthesis in *Pseudomonas* spp. bacteria. In addition, we observed genes in *T. hongkongensis* that are associated with secretion systems in other bacteria, including *clpV* (type VI secretory system), *epsE*, and *lspG* (type II secretory system). *T. hongkongensis* strains also contained *hemE* and *hemL* genes, which are observed in heme biosynthesis in *Haemophilus* spp. bacteria. 

Another species, *T. taiwanensis*, has previously been reported to produce alkaline protease and polyhydroxyalkanoates (PHA), renewable and biodegradable polymers that can replace conventional plastic (*6*). Comparison of *T. hongkongensis* and type strain *T. taiwanensis* LMG22826T (GenBank accession no. CP083911) showed that *T. hongkongensis* contained alkaline proteases, except lysyl endopeptidase. Furthermore, *T. taiwanensis* possesses *phaC* and *phaR* genes that are necessary for PHA production, as well as *phaZ* coding PHA depolymerase. Similarly, all 3 *T. hongkongensis* strains possessed the *phaC*, *phaR*, and *phaZ*, suggesting that *T. hongkongensis* is associated with PHA production. Oxidation of thiosulfate to sulfate has been reported in all *Tepidimonas* species through the *sox* pathway (*7*), and all 3 *T. hongkongensis* strains contained the genes encoding the sulfur-oxidizing protein.

Using the National Center for Biotechnology Information AMRfinder (https://www.ncbi.nlm.nih.gov/pathogens/antimicrobial-resistance/AMRFinder), we found that all 3 *T. hongkongensis* harbored aminoglycoside resistance genes (*aadA2*) and a β-lactamase gene (*bla*_OXA-2_). We also identified stress-related genes, including multiple heat-shock proteins and efflux pumps, including *qacL* (Appendix Table 2).

#### Characteristics for Patients with *T. hongkongensis* Strains

The isolate of strain HKU77^T^ was from a patient with pulmonary metastases. The DNA GC content was 67.2% mol; we considered this the type strain. The isolate of the second strain, HKU78, was from a patient with community-acquired pneumonia and had DNA GC content of 67.1% mol. The isolate of the third strain, HKU79, was from a patient with underlying Graves’ disease and paranoid schizophrenia and had DNA GC content of 67.2% mol.

### Environmental Sample Collection and Clinical Correlations

We collected 101 water samples from the faucets in various areas of the hospital, including wards, utility rooms, bathrooms, and the pantry, after the study period in April 2025. Among collected samples, 47 (46.5%) grew thermotolerant bacteria (Table 7). From those 47 samples, we subsequently identified 43 (91.5%; 42.6% of all water samples collected) thermotolerant bacteria as *Schlegelella aquatica*. We identified *T. hongkongensis* in 2 (2.0%) water samples and *Prophyrobacter cryptus* bacteria in 1 (1.0%) water sample. The percentages of water samples growing *S. aquatica* (42.6%) and *T. hongkongensis* (2.0%) were higher than the rates in respiratory specimens: 0.6% *S. aquatica* and 0.2% *T. hongkongensis* (p<0.05). Although we identified >20 times more *S. aquatica* isolates than *Tepidimonas* spp. isolates in the water samples, we isolated 3 times as many *Tepidimonas* spp. bacteria than *S. aquatica* in respiratory specimens. Furthermore, among the 17 patients with *Tepidimonas* spp. bacteria isolated from sputum 48 hours after hospitalization (Table 2), none were hospitalized in the 2 wards from which we isolated *T. hongkongensis* in the water samples.

**Table 7:** Bacteria from 101 water samples collected in hospital for study of novel thermotolerant *Tepidimonas* spp. bacteria in human respiratory specimens, Hong Kong, China, 2024*

Thermotolerant bacteria	No. (%) samples
*Schlegelella aquatica*	43 (42.6)
*Tepidimonas hongkongensis* sp. nov.	2 (2.0)
*Prophyrobacter cryptus*	1 (1.0)
Alphaproteobacterium	1 (1.0)

*Water samples were collected from faucets in various areas of the hospital, including wards, utility rooms, bathrooms, and the pantry.

## Discussion

Thermotolerant bacteria can thrive under elevated temperatures, typically 45°C–60°C. Examples of thermotolerant bacteria causing human infection include *Campylobacter fetus* (*8*) and *Mycobacterium xenopi* (*2*). Various thermotolerant bacteria, such as *Tepidimonas* spp., have been isolated or detected in environmental hot water samples, including hot springs (*9*–*11*). However, the clinical consequences of most thermotolerant bacteria are uncertain.

In addition to *Tepidimonas* spp., our study identified different thermotolerant bacteria in clinical specimens, including *S. aquatica*, which belongs to the Comamonadaceae family and was first isolated from a hot spring in Taiwan (*9*) but was not known to cause human infection. In addition, we identified *Vulcaniibacterium thermophilum* in 4 patient samples; it was first isolated from a geothermal soil sample in Tengchong, Yunnan Province, in southwest China in 2012 (*10*) and was the reported cause of prosthetic joint infection in 1 case (*13*). In addition, *Thermomonas* spp. bacteria, which we detected in 2 specimens, frequently have been isolated from hot water (*11*,*14*), marine sediment (*15*), and soil (*16*–*18*). One study reported its detection in the naso-oropharyngeal microbiome from breast cancer patients with severe COVID-19 (*19*). *Lactobacillus delbrueckii* bacteria, which we detected in 1 specimen, commonly are used for probiotics and food fermentation (*20*); however, that bacterium has been reported to cause urinary tract infection and bacteremia (*21*–*24*). Finally, we detected 3 *Bacillus* spp. bacteria, which are known to form heat-resistant spores; although non–*B.* *anthracis Bacillus* are usually considered environmental bacteria, they can cause infection in patients with prostheses and central catheters (*25*,*26*).

The genus *Tepidimonas* was established in 2000 (*27*), and its members are gram-negative bacilli with positive catalase and oxidase activity that are motile due to a single polar flagellum. *Tepidimonas* spp. bacteria typically exhibit optimal growth at temperatures >45°C; therefore, most have been isolated from relatively high-temperature environments, such as hot springs and spas worldwide (*9*–*11*). As of January 25, 2026, only 8 *Tepidimonas* species have been validly published in the List of Prokaryotic names with Standing in Nomenclature: *T. ignava*, *T. aquatica*, *T. taiwanensis*, *T. thermarum*, *T. fonticaldi*, *T. sediminis*, *T. alkaliphila*, and *T. charontis* (https://lpsn.dsmz.de/genus/tepidimonas). Within the genus, *T. taiwanensis* is the only strain that uses glucose and fructose for growth, due to the presence of putative ABC glucose/mannose (*gtsABCD*) and fructose (*frcABC*) transporters (*7*), whereas other species are frequently referred to as asaccharolytic. Similar to a previous report (*7*), the novel *T. hongkongensis* bacterium does not use glucose and fructose for growth. Of note, *T. taiwanensis* produces alkaline protease and PHA, renewable and biodegradable polymers that can replace conventional plastic (*6*). Genome analysis revealed that *T. hongkongensis* contains most of the alkaline protease genes found in *T. taiwanensis*, as well as genes associated with PHA production (*12*). Analysis of stress-related genes in *T. hongkongensis* revealed the presence of heat-resistant protein and ATPase genes, which explain its thermotolerant characteristic.

In our study, 0.6% of respiratory specimens grew *Tepidimonas* spp. bacteria after prolonged incubation at 50°C, and most patients from whom specimens were collected had solid organ malignancy and chronic lung disease, although ages were wide ranging. Other *Tepidimonas* spp. bacteria have been detected in clinical samples. For instance, a proposed new *Tepidimonas* species, *T. arfidensis*, was cultured from a bone marrow aspirate sample from a patient with leukemia with neutropenic fever (*28*). Other *Tepidimonas* spp. bacteria have been implicated in different human microbiome studies, including the urine microbiome for urinary incontinence (*29*,*30*), the endometrial microbiome for endometriosis (*31*), the gut microbiome in primary sclerosing cholangitis (*32*), and the sinus microbiome in chronic rhinosinusitis (*33*). In our study, only 36.6% of the patients had growth of other pathogenic bacteria in the sputum (Table 3), suggesting that *Tepidimonas* spp. are pathogenic and can cause lower respiratory tract infection. 

The tap water samples collected from the hospital revealed the presence of *T. hongkongensis* sp. nov. and a high percentage of *S. aquatica*. Those results align with previous findings that *Tepidimonas* spp. and *S. aquatica* bacteria are commonly found in the environment (*9*,*34*). Of note, we detected *Tepidimonas* spp. bacteria more frequently than *S. aquatica* in the water samples, but we detected more *Tepidimonas* spp. than *S. aquatica* in respiratory specimens (Tables 1, 7), suggesting that *Tepidimonas* spp. bacteria could have a greater potential for colonizing the human respiratory tract than other environmental bacteria. 

Comparative genomic analysis revealed that all 3 *T. hongkongensis* strains contained various virulence genes spanning critical functional classes, including adherence, iron uptake, lipid metabolism, nutritional virulence, serum resistance, and stress adaptation, but those genes are absent in *S. aquatica* LMG 23380 (Appendix Table 1). Those genes support colonization of host tissue, nutrient scavenging, immune evasion, and persistence under stress, further underscoring the potential pathogenicity of *T. hongkongensis.* Although resistome profiling did not demonstrate substantial antimicrobial resistance in *T. hongkongensis* (Appendix Table 2), we did detect it in environmental samples, and its ability to acquire other resistance, such as colistin resistance, through horizontal gene transfer from other gram-negative bacteria, is not known (*35*,*36*). Therefore, further surveillance is warranted.

Whether detection of *T. hongkongensis* in the hospital water systems warrants interventions requires further risk assessments and studies. First, the infectious threshold of *T. hongkongensis* should be established to assess the risk that patients could acquire it from the water system. Furthermore, whether usual water disinfection protocols, such as chlorine or ultraviolet light, are effective against this bacterium is unknown. Ongoing vigilance and documentation will help define the baseline incidence and clinical significance for this bacterium and guide future recommendations.

The first limitation of our study is that it was conducted in a hospital network in Hong Kong; whether similar observations occur in other hospitals and countries requires further investigation. Second, we only used 50°C for agar incubation to isolate thermotolerant bacteria, which might have failed to recover other clinically relevant pathogens that do not grow at such high temperatures. Third, although isolation of *Tepidimonas* spp. bacteria from the clinical specimens in our study might be related to a hot water source, other studies have isolated various *Tepidimonas* spp. bacteria in sterile clinical specimens, suggesting this genus might not solely be an environmental contaminant. Finally, isolation of *Tepidimonas* spp. and other thermotolerant bacteria in respiratory specimens could represent colonization instead of respiratory pathogens. However, many patients in our study had underlying solid organ malignancy, suggesting pathogenicity is still possible in severely immunocompromised patients, such as bone marrow transplant recipients. Further studies are required to investigate the pathogenic potential of *T. hongkongensis* and the range of illnesses the bacteria can cause.

In conclusion, we successfully isolated 42 thermotolerant *Tepidimonas* spp. bacterial strains from clinical specimens, including 3 novel strains confirmed by whole-genome sequencing. We propose the name *T. hongkongensis* sp. nov. to describe the 3 novel strains and designation of HKU77^T^ as the type strain of this novel species. The combination of high incubation temperature for selection, prolonged incubation for growth, and MALDI-TOF mass spectrometery and 16S rRNA sequencing enabled discovery of this novel species. Analysis of water samples from the hospital further confirmed the presence of *T. hongkongensis* in the environment. Although clinical implications of *T. hongkongensis*, especially in immunocompromised patients, are not yet known, its potential spread in water systems is concerning. Ongoing vigilance and documentation will help define the baseline incidence and clinical significance of *T. hongkongensis* and guide future recommendations. 

Appendix Additional information on detection of novel thermotolerant *Tepidimonas* species bacteria in human respiratory specimens, Hong Kong, China, 2024.
