# Correction: Vol. 32, No. 1

**DOI:** 10.3201/eid3202.C13202

**Published:** 2026-02

**Keywords:** errata, erratum, correction

Essential taxonomic information was inadvertently omitted from Detection of Novel Thermotolerant *Tepidimonas* sp. Bacteria in Human Respiratory Specimens, Hong Kong, China, 2024 (K.H.-Y. Chiu et al.). The article has been corrected online (https://wwwnc.cdc.gov/eid/article/32/1/25-0818_article).

