# Supplementary material for: Detection of Novel Thermotolerant Tepidimonas sp. Bacteria in Human Respiratory Specimens, Hong Kong, China, 2024
Source: Emerg Infect Dis. 2026 Jan;32(1):82–92. doi: 10.3201/eid3201.250818 (PMC12870156; doi:10.3201/eid3201.250818)
Supplement: Appendix — Additional information on detection of novel thermotolerant Tepidimonas species bacteria in human respiratory specimens, Hong Kong, China, 2024. [file 25-0818-Techapp-s1.pdf]

Article DOI: https://doi.org/10.3201/eid3201.250818

EID cannot ensure accessibility for supplementary materials supplied by authors. Readers who have difficulty accessing supplementary content should contact the authors for assistance.

Detection of Novel Thermotolerant *Tepidimonas* Species Bacteria in Human Respiratory Specimens, Hong Kong, China, 2024

Appendix

Methods

Partial 16S rRNA sequencing

For partial 16S rRNA sequencing, genomic DNA extraction was performed using the QIAamp DNA Mini Kit according to the manufacturer's instructions (QIAGEN). Two microliters of DNA extract was added with 23 µL PCR reaction mix, which includes 10X concentrated PCR buffer (100 mM Tris-HCl, pH 8.3, 500 mM KCl), 25 mM $MgCl_2$ solution, 100 µM forward primer 16S-1F (AGTTTGATCMTGGCTCAG), 100 µM reverse primer 16S-2R (GGACTACHAGGGTATCTAAT), dNTPs (100 mM dGTP, 100 mM dATP, 100 mM dTTP, and 100 mM dCTP) and AmpliTaq Gold DNA Polymerase (5U/µL) from Applied Biosystems. Thermocycling was performed with an initial activation at 95°C for 10 minutes, followed by 40 cycles of 94°C for 1 minute, 48°C for 1 minute, and 72°C for 1 minute. Purification was performed using the QIAquick PCR Purification Kit (QIAGEN). Cycle sequencing used 3 µL of purified PCR product with 17 µL master mix (4 µL of 5× sequencing buffer, 1 µL BigDye Terminator mix, 3.2 µM primer and nuclease-free H_2O). Thermocycling included 25 cycles of 96°C for 10 seconds, 50°C for 5 seconds and elongation at 60°C for 4 minutes. Samples were sequenced on the Applied Biosystems 3500 Genetic Analyzer. Results were analyzed using the NCBI BLAST database.

Phenotypic characterization of novel bacterial species

Further phenotypic characterization was performed for bacterial isolates that cannot be accurately identified through 16S rRNA sequencing (i.e., novel species). Gram staining was performed on the bacterial colonies. The morphological characteristics were determined on horse blood agar at 50°C. Motility was checked using the wet mount method. Culture characteristics were tested on blood agar at 25°C, 37°C, 42°C, and 50°C for 72 hours, and on chocolate agar, Hemophilus test medium (HTM) agar, Brucella agar, Buffered charcoal yeast extract (BCYE) agar, and Brain heart infusion (BHI) agar with supplementation of X and V factor at 50°C for 72 hours. Activities of catalase, oxidase, urease, nitrate, DNase, lipase, gelatinase, lysine decarboxylase were determined, and sugar fermentation (glucose, lactose, sucrose, citrate), indole, H_2S production, and ortho-Nitrophenyl-β-galactoside (ONPG) were performed according to our laboratory standard operating procedure. The biochemical tests were incubated at 50°C when overnight incubation was necessary. The VITEK AMS GNI card (bioMérieux) was read automatically by the instrument within 24 hours. UV fluorescence was tested by subjecting the bacterial colonies to UV light of wavelength 254 nm for 5 minutes and looking for fluorescence in a dark environment. Antimicrobial susceptibility testing was performed by the disk diffusion method and the E-test (for penicillin MIC and vancomycin MIC) on Mueller-Hinton agar at 50°C, read at 72 hours. Results were interpreted according to the Clinical and Laboratory Standards Institute (CLSI) *Pseudomonas* breakpoint (*1*).

Whole-genome sequencing

Whole-genome sequencing was performed on all the novel species identified. A dual-platform approach was employed for whole genome sequencing. This involved using Illumina short-read sequencing and Oxford Nanopore Technologies (ONT) long-read sequencing for high-resolution genotyping. Genomic DNA preparation was initially performed using the Nextera DNA Prep Kit, followed by sequencing on the Illumina iSeq100 platform to generate short reads. Trimmomatic is used to trim and remove adapters and low-quality sequences. This involves specifying adaptor sequences to remove, automatically handling standard Illumina adaptors, and setting a minimum length threshold to discard reads shorter than 75 bp, thus retaining only high-quality, meaningful data. For long reads, genomic DNA was sheared using the Covaris g-TUBE and libraries prepared with the use of the Ligation Sequencing Kit (SQK-LSK109) and the Native Barcoding Expansion kits 1–12 (EXP-NBD104). Sequencing was

conducted using the Oxford Nanopore MinION device. Porechop is used to remove adaptors and possibly barcodes, ensuring that the actual sequence data are processed free from sequencing adapters or multiplexing barcodes. Subsequently, Filtlong filters out long reads based on quality and length, discarding any reads shorter than 1,000 bp to focus on longer, more informative reads. De novo hybrid assembly was performed using Unicycler version 0.5.0, which utilizes short reads to create the draft genome of contiguous sequences (contigs), then long reads to join the contigs to a complete genome with subsequent assembly polishing (*2*).

Phylogenetic and genomic analysis

The whole genome was annotated with Prokka and PGAP v2024–07–18.build 7555 (*3*,*4*). Core genome-based phylogenetic analysis was conducted using PhyloPhIan v.3.1.68 (*5*). By default, PhyloPhIan retrieves species-specific core genes from among >18,000 sets of preselected UniRef90 gene families. However, for highly diverse genomes, it switches to an alternative method employing the 400 most universal marker genes, coupled with aggressive alignment trimming parameters to improve alignment quality. The resulting multiple sequence alignments were then analyzed using IQ-Tree v.2.2.6 for phylogenetic inference; this involved model selection and bootstrapping to assess branch robustness. Finally, the phylogenetic tree was visualized and annotated in FigTree v.1.4.4 to visualize the evolutionary relationships among the isolates. Furthermore, the genes of the novel species were further identified using different software. Virulence factors were identified by VFAnalyzer using the Virulence Factors of Pathogenic Bacteria database (VFDB). AMR and stress-related genes were identified using NCBI AMRfinder (*6*). Functional annotation was performed using Rapid Annotations using Subsystems Technology (RAST) (*7*). The Type Strain Genome Server (TYGS) was used to calculate digital DNA-DNA hybridization (dDDH) values between the genomes of *Tepidimonas* using d4 formula with a threshold value of 70.0% for species delineation (*8*). A genome-based phylogenetic tree was constructed to determine the phylogenetic relationships among the studied strains and the type strain of the existing *Tepidimonas* species (*8*).

Environmental sample collection

In view of the close relationship between *Tepidimonas* and water, water samples were collected from the taps of different wards in Queen Mary Hospital in April 2025. Water samples were inoculated into the labeled sterile bottle with nutrient broth (Oxoid CM67, containing meat extract, peptone, and sodium chloride) with and without supplementation of vancomycin at a

final concentration of 10 μg/mL. The inoculated broths were then incubated at 50°C for 5 days, and blind subculture was performed on blood agar plates, with the plates incubated at 50°C for 2 days. Any colonies that grew from the blood agar plates at 50°C were subjected to subsequent identification as described above using MALDI-TOF MS, then 16S rRNA PCR if MALDI-TOF MS fails.

References

1. Clinical and Laboratory Standards Institute (CLSI). Performance standards for antimicrobial susceptibility testing: thirty-fourth informational supplement (M100–ED34), Wayne (PA): The Institute;2024.

2. Wick RR, Judd LM, Gorrie CL, Holt KE. Unicycler: Resolving bacterial genome assemblies from short and long sequencing reads. PLOS Comput Biol. 2017;13:e1005595. PubMed https://doi.org/10.1371/journal.pcbi.1005595

3. Seemann T. Prokka: rapid prokaryotic genome annotation. Bioinformatics. 2014;30:2068–9. PubMed https://doi.org/10.1093/bioinformatics/btu153

4. Tatusova T, DiCuccio M, Badretdin A, Chetvernin V, Nawrocki EP, Zaslavsky L, et al. NCBI prokaryotic genome annotation pipeline. Nucleic Acids Res. 2016;44:6614–24. PubMed https://doi.org/10.1093/nar/gkw569

5. Asnicar F, Thomas AM, Beghini F, Mengoni C, Manara S, Manghi P, et al. Precise phylogenetic analysis of microbial isolates and genomes from metagenomes using PhyloPhlAn 3.0. Nat Commun. 2020;11:2500. PubMed https://doi.org/10.1038/s41467-020-16366-7

6. Feldgarden M, Brover V, Haft DH, Prasad AB, Slotta DJ, Tolstoy I, et al. Validating the AMRFinder tool and resistance gene database by using antimicrobial resistance genotype-phenotype correlations in a collection of isolates. Antimicrob Agents Chemother. 2019;63: e00483-19. PubMed https://doi.org/10.1128/AAC.00483-19

7. Aziz RK, Bartels D, Best AA, DeJongh M, Disz T, Edwards RA, et al. The RAST server: rapid annotations using subsystems technology. BMC Genomics. 2008;9:75. PubMed https://doi.org/10.1186/1471-2164-9-75

8. Meier-Kolthoff JP, Göker M. TYGS is an automated high-throughput platform for state-of-the-art genome-based taxonomy. Nat Commun. 2019;10:2182. PubMed https://doi.org/10.1038/s41467-019-10210-3

Appendix Table 1. Virulence factors identified in the 3 *T. hongkongensis* and *S. aquatica* through the Virulence Factors of Pathogenic Bacteria database (VFDB).

Virulence factor classes	Virulence factors	Related genes	Patient strains HKU77[T]	HKU78	HKU79	*S. aquatica* LMG 23380[T]
Adherence	Type IV pili	*pilD*	Yes	Yes	Yes	Yes
	Type IV pili biosynthesis (*Pseudomonas*)	*pilB*	Yes	Yes	Yes	Yes
		pilC	Yes	Yes	Yes	–
		pilR	Yes	Yes	Yes	–
		pilT	Yes	Yes	Yes	Yes
	Type IV pili (*Neisseria*)	*pilT2*	Yes	Yes	Yes	Yes
	Alginate biosynthesis (*Pseudomonas*)	*algC*	Yes	Yes	Yes	Yes
	Alginate regulation (*Pseudomonas*)	*algU*	Yes	Yes	Yes	Yes
		algW	Yes	Yes	Yes	Yes
	Capsular polysaccharide (*Vibrio*)	*rmlB*	Yes	Yes	Yes	Yes
		wbfV/wcvB	Yes	Yes	Yes	–
	Capsule (*Klebsiella*)	*uge*	Yes	Yes	Yes	–
Invasion	Flagella	*cheA*	Yes	Yes	Yes	Yes
		cheB	Yes	Yes	Yes	Yes
		cheD	Yes	Yes	Yes	Yes
		cheR	Yes	Yes	Yes	Yes
		cheW	Yes	Yes	Yes	Yes
		cheY1	Yes	Yes	Yes	Yes
		cheY	Yes	Yes	Yes	Yes
		flgC	Yes	Yes	Yes	Yes
		flgG	Yes	Yes	Yes	Yes
		flgI	Yes	Yes	Yes	Yes
		flhA	Yes	Yes	Yes	Yes
		fliA	Yes	Yes	Yes	Yes
		fliF	Yes	Yes	Yes	Yes
		fliG	Yes	Yes	Yes	Yes
		fliI	Yes	Yes	Yes	Yes
		fliM	Yes	Yes	Yes	Yes
		fliN	Yes	Yes	Yes	Yes
		fliP	Yes	Yes	Yes	Yes
		fliQ	Yes	Yes	Yes	Yes
		motA	Yes	Yes	Yes	Yes
		motB	Yes	Yes	Yes	Yes
		tsr	Yes	Yes	Yes	Yes
Secretion system	T6SS-1	*clpV*	Yes	Yes	Yes	Yes
	Flagella (cluster I) (*Yersinia*)	*flhC*	Yes	Yes	Yes	Yes
	T4SS effectors (*Coxiella*)		Yes	Yes	Yes	Yes
			Yes	Yes	Yes	Yes
Amino acid and purine metabolism	Purine synthesis (*Mycobacterium*)	*purC*	Yes	Yes	Yes	–
Endotoxin	LOS (*Hemophilus*)	*lpxC*	Yes	Yes	Yes	Yes
Immune evasion	Capsule (*Acinetobacter*)		Yes	Yes	Yes	Yes
	Exopolysaccharide (*Hemophilus*)	*mrsA/glmM*	Yes	Yes	Yes	Yes
	Polysaccharide capsule (*Bacillus*)	*galE*	Yes	Yes	Yes	Yes
Iron uptake	Heme biosynthesis (*Hemophilus*)	*hemE*	Yes	Yes	Yes	–
		hemL	Yes	Yes	Yes	Yes
Lipid and fatty acid metabolism	Isocitrate lyase (*Mycobacterium*)	*icl*	Yes	Yes	Yes	–
Motility	Flagella (*Bordetella*)	*flhD*	Yes	Yes	Yes	–
Nutritional virulence	Biotin metabolism (*Francisella*)		Yes	Yes	Yes	–
Others	O-antigen (*Yersinia*)		Yes	Yes	Yes	Yes
Serum resistance	LPS rfb locus (*Klebsiella*)	*rmlC*	Yes	Yes	Yes	–
		rmlD	Yes	Yes	Yes	Yes
Serum resistance and immune evasion	Capsule (*Francisella*)	*rpe*	Yes	Yes	Yes	Yes
Stress adaptation	Catalase-peroxidase (*Mycobacterium*)	*katG*	Yes	Yes	Yes	–
	Manganese transport system (*Neisseria*)	*mntA*	Yes	Yes	Yes	–
		mntB	Yes	Yes	Yes	–

*Yes, presence of the virulence factor; –, absence of the virulence factor.

Appendix Table2. Antimicrobial resistance (AMR) and stress-related genes in 3 strains of *T. hongkongensis* isolated in this study*

Gene	Description	HKU77^T	HKU78	HKU79
AMR				
aadA2	Targeting aminoglycoside	Yes	Yes	Yes
*bla*OXA-2	Targeting β-lactam	Yes	Yes	Yes
Stress related				
qacL	Quaternary ammonium efflux	Yes	Yes	Yes
clpK	Heat shock ATPase	Yes	Yes	Yes
hdeD-GI	Heat resistance membrane protein	Yes	Yes	Yes
shsP	Small heat shock protein	Yes	Yes	Yes
trxLHR	Heat resistance thioredoxin	Yes	Yes	Yes
yfdX1	Heat resistance protein	Yes	Yes	Yes
yfdX2	Heat resistance protein	Yes	Yes	Yes

* Yes, presence of the gene. AMR, antimicrobial resistance.

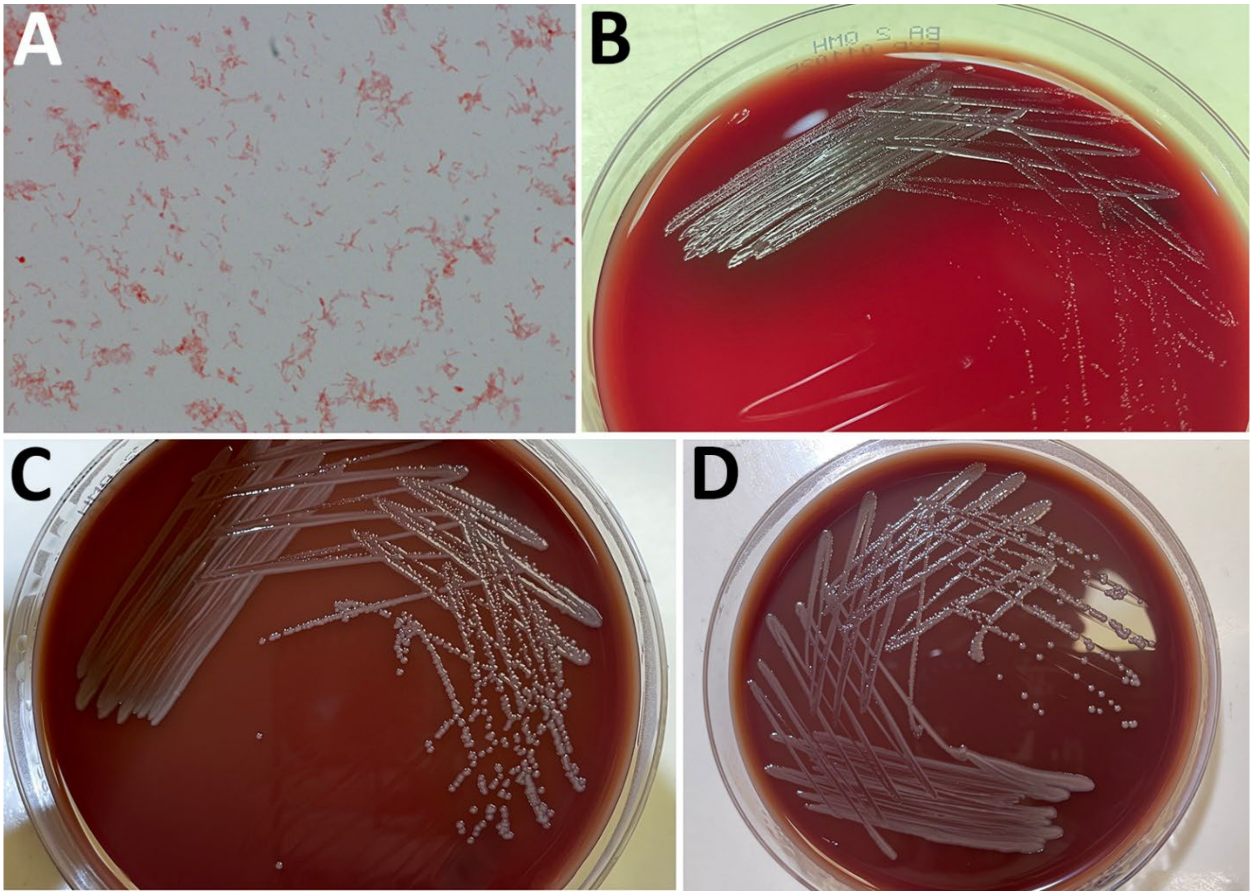

Appendix Figure. Gram stain and colonization of *Tepidimonas* species detected in human respiratory samples, Hong Kong, China. A) Gram stain of *T. hongkongensis* sp. nov. showing gram-negative, nonspore forming rods with occasional spherical enlargement. B–D) Blood agar colony morphology of *T. hongkongensis* sp. nov. after 72 hours at 35°C (B), 42°C (C), and 50°C (D).
